# New ZrO_2_/Al_2_O_3_ Nanocomposite Fabricated from Hybrid Nanoparticles Prepared by CO_2_ Laser Co-Vaporization

**DOI:** 10.1038/srep20589

**Published:** 2016-02-05

**Authors:** José F. Bartolomé, Anton Smirnov, Heinz-Dieter Kurland, Janet Grabow, Frank A. Müller

**Affiliations:** 1 Instituto de Ciencia de Materiales de Madrid (ICMM), Consejo Superior de Investigaciones Científicas (CSIC), C/Sor Juana Inés de la Cruz 3, 28049 Madrid, Spain; 2 Friedrich Schiller University Jena, Otto Schott Institute of Materials Research (OSIM), Löbdergraben 32, 07743 Jena, Germany

## Abstract

Alumina toughened zirconia (ATZ) and zirconia toughened alumina (ZTA) are currently the materials of choice to meet the need for tough, strong, and bioinert ceramics for medical devices. However, the mechanical properties of ZrO_2_/Al_2_O_3_ dispersion ceramics could be considerably increased by reducing the corresponding grain sizes and by improving the homogeneity of the phase dispersion. Here, we prepare nanoparticles with an intraparticular phase distribution of Zr_(1−x)_Al_x_O_(2−x/2)_ and (γ-, δ-)Al_2_O_3_ by the simultaneous gas phase condensation of laser co-vaporized zirconia and alumina raw powders. During subsequent spark plasma sintering the zirconia defect structures and transition alumina phases transform to a homogeneously distributed dispersion of tetragonal ZrO_2_ (52.4 vol%) and α-Al_2_O_3_ (47.6 vol%). Ceramics sintered by spark plasma sintering are completely dense with average grain sizes in the range around 250 nm. Outstanding mechanical properties (flexural strength σ_*f*_ = 1500 MPa, fracture toughness *K*_*Ic*_ = 6.8 MPa m^1/2^) together with a high resistance against low temperature degradation make these materials promising candidates for next generation bioceramics in total hip replacements and for dental implants.

Composites of the oxide ceramics α-alumina (α-Al_2_O_3_) and tetragonal zirconia (t-ZrO_2_) are used in numerous technical and biomedical applications due to their combination of excellent corrosion resistance, high electrical resistivity, good biocompatibility, low friction, high wear resistance, and high strength^1^. In particular for biomedical applications like dental implants and total joint prostheses their combination is a material of choice if highly reliable biomaterials with excellent mechanical properties are required^2^.

A dispersion of both materials in so-called alumina toughened zirconia (ATZ) and zirconia toughened alumina (ZTA) aims on combining the high strength and toughness of t-ZrO_2_ with the excellent tribological properties and ageing resistance of α-Al_2_O_3_^1^,^2^. Beyond, small amounts of alumina are known to stabilize the tetragonal ZrO_2_ matrix phase^3^ and to inhibit grain growth by Zener pinning^4^. However, the mechanical properties of ZrO_2_/Al_2_O_3_ dispersion ceramics described in literature differ by a huge amount because they depend to a large extend on the ratio and homogeneity of the phase distribution, on the grain size and porosity, and on processing as well as sintering conditions^1^,^2^,^5^,^6^,^7^,^8^. Exemplarily, the flexural strength σ_*f*_ and fracture toughness *K*_*Ic*_ of state of the art ZrO_2_/Al_2_O_3_ dispersion ceramics described in literature reach up to 1288 MPa and 6.2 MPa m^1/2^ for ZTA ceramics^7^ consisting of 76 mass% Al_2_O_3_ (average grain size *d*_*50*_ = 730 nm) and 24 mass% ZrO_2_ (*d*_*50*_ = 330 nm), respectively, and 1166 MPa and 7.4 MPa m^1/2^ for ATZ ceramics^2^ consisting of 80 mass% ZrO_2_ and 20 mass% Al_2_O_3_ with *d*_*50*_ values around 400 nm for both components. It is known that the mechanical properties of ZrO_2_/Al_2_O_3_ composites can be considerably increased by reducing the corresponding grain sizes and by improving the homogeneity of the phase dispersion^9^. Furthermore, these factors are critical under hydrothermal conditions. In ZTA ceramics the spontaneous transformation of tetragonal to monoclinic ZrO_2_ grains in a humid environment and in the temperature range 20 °C to 300 °C, i.e. low temperature degradation (LTD), occurs more readily if the grain size is below a critical value (500 nm)^10^. Additionally, their narrow size distribution as well as their concentration and isolation, i.e. the absence of aggregates, are important for the inhibition of intergranular water diffusion which would lead to a premature transformation^10^. For ATZ the dispersion and size distribution of the Al_2_O_3_ grains are even more critical for the LTD behaviour because of the large proportion of t-ZrO_2_^11^. For this purpose ZrO_2_/Al_2_O_3_ nanoparticles with an intraparticular phase dispersion instead of just mixing different portions of raw materials might be desirable.

In order to synthesize these special hybrid nanoparticles the highly flexible and versatile CO_2_ laser co-vaporization (CoLAVA) process was used^12^. Starting material is a homogenous mixture of coarse-grained ceramic raw powders which are co-vaporized in the intense focus of a CO_2_ laser beam. Subsequent rapid cooling induces the simultaneous condensation of the components resulting in the formation of composite nanoparticles in a continuously running, scalable^13^ process. In general, the CoLAVA nanoparticles are spherically shaped, narrowly size-distributed, crystalline, and merely softly agglomerated by weak van der Waals forces. In contrast to other synthesis routes especially designed precursors are not required because the chemical composition of the ceramic starting powders corresponds to that of the desired composite nanopowders. Thus, contaminations of the nanopowders by reaction by-products are excluded.

Recently, we demonstrated that the CoLAVA method in combination with subsequent spark plasma sintering (SPS) is highly suitable to prepare 2 mol% Y_2_O_3_ (yttria) stabilized t-ZrO_2_ ceramics with a flexural strength of 1380 MPa, a fracture toughness of 13 MPa m^1/2^, and a high resistance against LTD^14^. In our present work we now used a homogeneous mixture of yttria stabilized zirconia and alumina raw powders to synthesize a ZrO_2_/Al_2_O_3_ composite nanopowder by CoLAVA. We investigated phase transformations during thermal treatment of the powders and evaluated the homogeneity of the phase distribution after SPS. Mechanical properties and LTD behaviour of the resulting composite ceramics were characterized.

## Results

### CoLAVA nanoparticles

**Figure 1:**
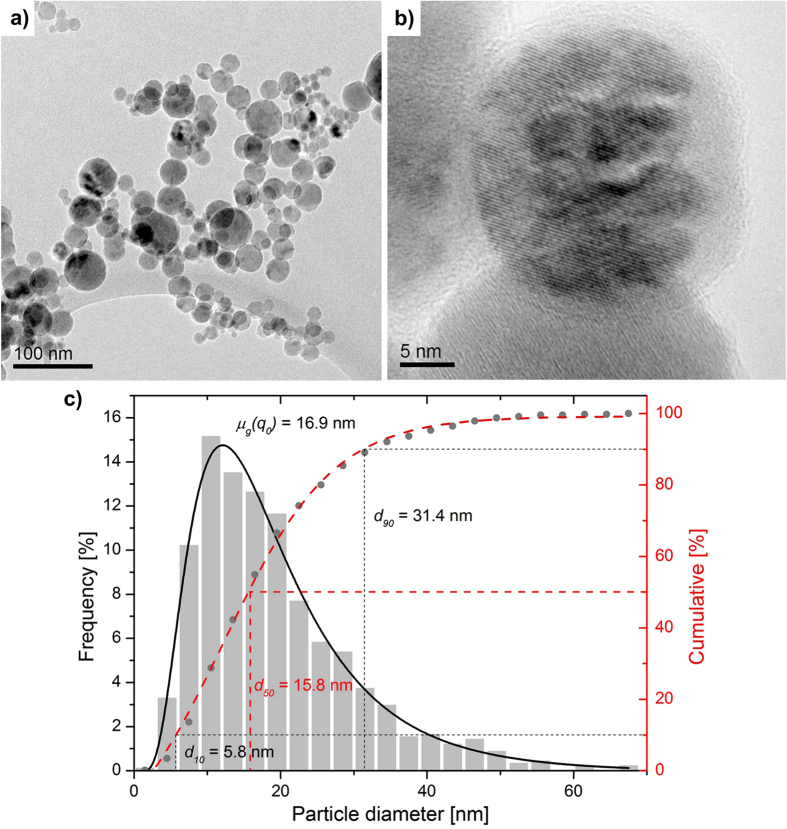
TEM investigation of the CoLAVA nanoparticles. (**a**) survey and (**b**) high resolution micrographs, (**c**) frequency based particle diameter distribution of the CoLAVA nanopowder (log normal (**—**) and cumulative (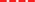) distribution) with the geometric mean diameter *μ*_*g*_*(q*_0_) and the characteristic diameters *d*_*10*_, *d*_*50*_, and *d*_*90*_.

Figure 1 shows transmission electron microscopy (TEM) images and the size distribution of the hybrid nanoparticles obtained from CoLAVA of the Al_2_O_3_/Y_2_O_3_-ZrO_2_ raw powder mixture. The particles are spherically shaped (Fig. 1a), and their diameters follow a log normal distribution (Fig. 1c) with an average diameter *d*_*50*_ of 15.8 nm and a specific surface area *S*_*BET*_ of 49.7 m^2 ^g^−1^. The particles appear crystalline with visible lattice planes. However, in high resolution micrographs some of the particles show a core/shell structure (Fig. 1b) where the core consists of crystalline phases and the shell seems to be amorphous. The thickness of the shell is generally below 1 nm and reaches up to 5 nm in very few cases as illustrated in Fig. 1b. The rate of production of the Al_2_O_3_/ZrO_2_ nanopowder was 10.2 g h^−1^ under the applied CoLAVA process conditions. Image analyses of SEM micrographs of specimens sintered from the CoLAVA nanopowder revealed that its alumina content (37.4 mass%) exceeds the one of the raw powder mixture (20 mass%). This is due to differing rates of vaporization of alumina and zirconia in the raw powder mixture because of their different melting and vaporization temperatures *T*_*m*_ and *T*_*b*_, respectively (Al_2_O_3_: *T*_*m*_ = 2015 °C, *T*_*b*_ = 2980 °C; ZrO_2_: *T*_*m*_ = 2700 °C, *T*_*b*_ = 5155 °C), absorption coefficients at the CO_2_ laser wavelength (Al_2_O_3_: 3556 cm^−1^; ZrO_2_: 1185 cm^−1^)^15^,^16^, and thermal conductivities (Al_2_O_3_: 37.0 W m^−1 ^K^−1^ at 25 °C, 5.7 W m^−1 ^K^−1^ at 1600 °C; ZrO_2_: 1.7 W m^−1^ K^−1^ at 25 °C, 2.3 W m^−1 ^K^−1^ at 1600 °C)^17^. Therefore, it is not possible to map the mixing ratio of the raw components onto the phase composition of the resulting nanopowder. In order to obtain defined phase ratios in the nanopowder, the mixing ratio of the raw powders has to be determined experimentally. However, at this point it is important to mention that the reproducibility of the CoLAVA method is very high. Under the same experimental conditions (e.g. raw powder ratio, laser parameters, and process gas flows) the obtained results (e.g. composition, phase distribution, particle size and size distribution) are always exactly the same.

### Thermal behaviour of the CoLAVA nanopowder

**Figure 2:**
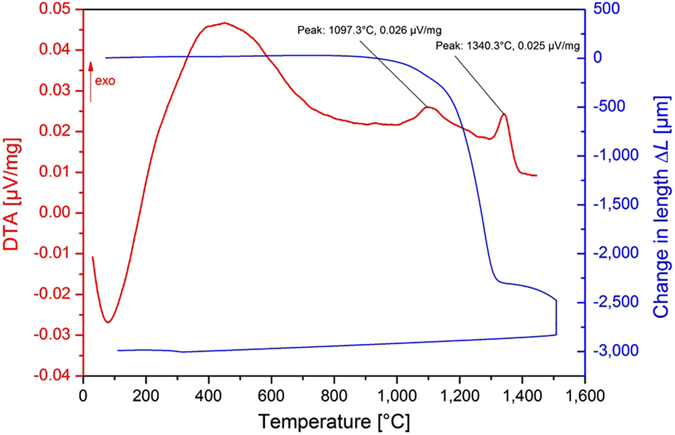
Thermoanalyses of the CoLAVA nanopowder. Differential thermal (DTA 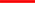) and dilatometric measurements (absolute change in length 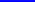).

Heating the CoLAVA nanopowder to 1445 °C results in two exothermic peaks in the differential thermal analysis (DTA) curve at 1097 °C and 1340 °C (Fig. 2). The dilatometry curve (Fig. 2) reveals that under conventional conditions the powder starts to sinter at a temperature around 900 °C and reaches its maximum sintering rate above 1200 °C. Above 1300 °C the densification slows down immediately, and the shrinkage rate drops to a minimum. After sintering at 1500 °C for 2 h the samples reached 88% of their theoretical density *φ*_*th*_ which is equivalent to a porosity of 12%.

**Figure 3:**
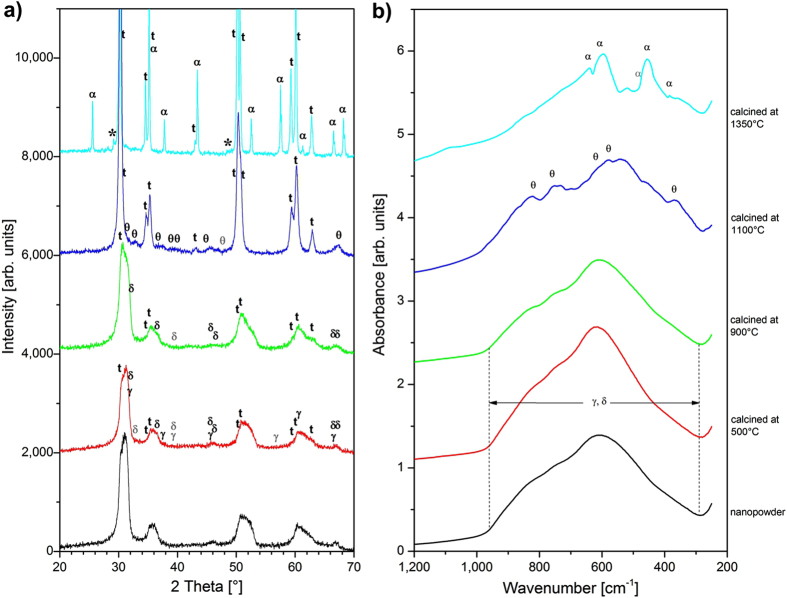
Evolution of the crystal phases in the CoLAVA nanopowder with increasing calcination temperatures. (**a**) XRD analyses and (**b**) FTIR spectrometry of the nanopowder as prepared (—) as well as calcined at 500 °C (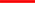), 900 °C (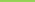), 1100 °C (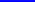), and 1350 °C (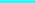), labelling “t” denotes tetragonal zirconia, “γ”, “δ”, “θ”, and “α” denote the alumina phases, and “*” marks yttria reflections.

The as prepared CoLAVA nanopowder consists of tetragonal zirconia (Fig. 3a) and amorphous or low crystalline transition alumina phases like γ-Al_2_O_3_ or δ-Al_2_O_3_ (Fig. 3b). The domain size *d*_*(101)*_ of t-ZrO_2_ calculated from the Scherrer equation amounts to 5 nm. The X-ray diffraction (XRD) reflections of t-ZrO_2_ (Fig. 3a) are slightly shifted towards higher diffraction angles 2*θ*. Heating the powder to 500 °C and 900 °C, respectively, has no influence on the composition or on the domain size. Heating the powder to 1100 °C which is the temperature of the first exothermic peak in the DTA curve (Fig. 2) results in a phase transition of the γ- and δ-alumina phases to θ-Al_2_O_3_ (Fig. 3b). The t-ZrO_2_ domains grow to *d*_*(101)*_ = 19 nm and the XRD reflections (Fig. 3a) shift back to the original angular positions of t-ZrO_2_ found in the Powder Diffraction File (PDF) 01-083-0113 from the International Centre for Diffraction Data (ICDD). At a temperature of 1350 °C which is in the range of the second exothermic peak in the DTA curve (Fig. 2) θ-Al_2_O_3_ transforms to highly crystalline α-Al_2_O_3_ (Fig. 3a,b) with a domain size of *d*_*(10-2)*_ = 49 nm, and the domains of t-ZrO_2_ grow to *d*_*(101)*_ = 48 nm. Moreover, at 1350 °C small additional reflections appear at 2*θ* angles of 29.2° and 48.6°. They represent the two most intense reflections of yttria and were assigned to its (222) and (440) planes, respectively, according to the ICDD-PDF 00-41-1105. Inductively coupled plasma-optical emission spectroscopy (ICP-OES) analyses reveal that CoLAVA nanoparticles calcined at 1350 °C consist of 60.4 ± 0.6 mass% ZrO_2_, 37.5 ± 0.3 mass% Al_2_O_3_, 1.3 ± 0.03 mass% Y_2_O_3_, and 0.8 ± 0.02 mass% HfO_2_. The latter represents an impurity that is usually present in ZrO_2_ raw powders.

### Composition, microstructure, mechanical properties, and low temperature degradation resistance of sintered Al_2_O_3_/ZrO_2_ dispersion ceramics

**Figure 4:**
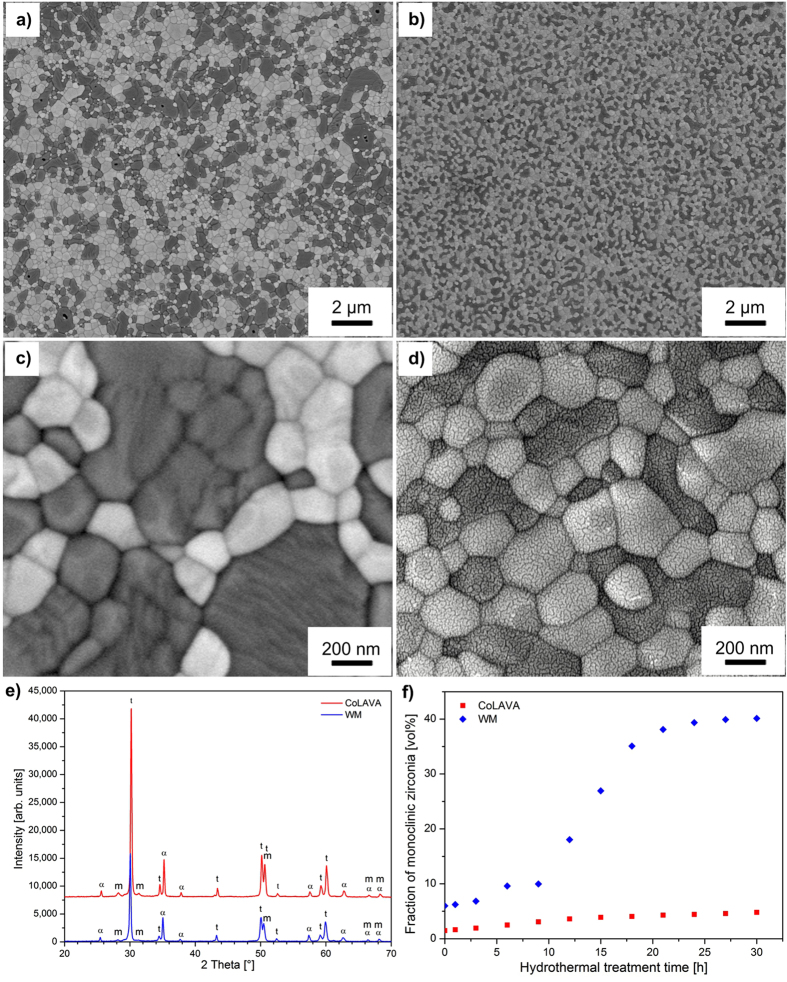
Ceramics sintered by SPS from the WM reference powder and from the CoLAVA nanopowder. SEM micrographs of polished and subsequently thermally etched surfaces (**a**,**c**) WM ceramic, (**b,d**) CoLAVA ceramic (bright phase t-ZrO_2_, dark phase α-Al_2_O_3_), (**e**) XRD analyses of polished surfaces (labelling “t” and “m” denote tetragonal and monoclinic zirconia, respectively, labelling “α” denotes α-alumina), and (**f**) LTD – evolution of the volume fraction of monoclinic transformed zirconia in dependence of the aging treatment time.

Figure 4e shows diffractograms obtained from polished surfaces of ceramics sintered from the wet mechanically mixed Al_2_O_3_/ZrO_2_ reference powder (WM) and from the CoLAVA nanopowder by SPS (3 min at 1400 °C and 80 MPa). The diffraction patterns are almost identical, and they reveal that the surfaces of both specimens mainly consist of tetragonal zirconia, α-alumina, and a minor amount of monoclinic zirconia. The scanning electron microscope (SEM) images in Fig. 4a–d show the microstructure of the sintered Al_2_O_3_/ZrO_2_ specimens. The specimen sintered from the WM powder (Fig. 4a,c) which was wet mechanically mixed from Al_2_O_3_ and ZrO_2_ powders in a 37.4:62.6 mass ratio had a density of 98% φ_*th*_. The specimen sintered from the CoLAVA nanopowder (Fig. 4b,d) which was prepared from a powder mixture of Al_2_O_3_ and ZrO_2_ in a 20:80 mass ratio comprised 37.4 mass% (i.e. 47.6 vol%) α-Al_2_O_3_ and 62.6 mass% (i.e. 52.4 vol%) t-ZrO_2_ and reached a density of 99% φ_*th*_. In both cases both phases are clearly separated. However, the grain sizes of both specimens differ. In the WM composites the average grain sizes of ZrO_2_ and Al_2_O_3_ were 403 ± 3 nm and 981 ± 5 nm, respectively, whereas they were 216 ± 2 nm and 270 ± 3 nm in the CoLAVA composites, respectively. Beyond that, the dispersion of the ZrO_2_ and Al_2_O_3_ grains in the CoLAVA composite (Fig. 4b) is much more homogeneous compared with the WM composite (Fig. 4a). Therein both the ZrO_2_ grains and also the Al_2_O_3_ grains are clustered together forming voluminous aggregates with maximum sizes exceeding 2 μm. Semi-quantitative microanalyses using energy dispersion spectroscopy (EDS) were conducted to determine the mean yttria content of the ZrO_2_ grains in the sintered specimens. It was found that the ZrO_2_ grains in the CoLAVA ceramic contain significantly less yttria (≈0.5 mol%) than in the case of the WM ceramic (≈2 mol%). On the other hand, in a recent study we have demonstrated that the homogeneity of the yttria distribution in ZrO_2_ ceramics depends on the type of preparation of the Y_2_O_3_/ZrO_2_ starting powder mixture. So a wet mechanically mixed starting powder generally leads to a less homogeneous yttria distribution when compared with a CoLAVA nanopowder prepared from the same conventionally mixed starting powder^14^. The superior homogeneity of the CoLAVA composite is also reflected in its corresponding mechanical properties (Table 1). The elastic modulus *E* of both specimens is in the same range. However, flexural strength σ_*f*_, Vickers hardness *HV*, and fracture toughness *K*_*Ic*_ of the CoLAVA composite significantly exceed the ones of the WM composite by 36%, 8%, and 45%, respectively, and reaches levels of 1500 MPa, 14.9 GPa, and 6.8 MPa m^1/2^, respectively. The volume fractions of monoclinic zirconia (Table 1) on polished and on fractured surfaces of the ceramic specimens were calculated from XRD data using equations (1) and (2). It was found that the transformability *V*_*trans*_ of t-ZrO_2_, determined as the difference of the contents of m-ZrO_2_ in the polished and in the fractured surfaces of the specimens, is around 34% for the CoLAVA ceramic and only 9% for the WM ceramic (Table 1).

The LTD resistance of sintered specimens was evaluated under hydrothermal conditions (Fig. 4f). After 30 h at 134 °C the volume fraction of monoclinic zirconia increased from 2% to 5% in the CoLAVA ceramic and from 6% to 40% in the WM ceramic.

## Discussion

It was shown that the laser co-vaporization of Al_2_O_3_ and ZrO_2_ powders in a homogeneous mixture is a very suitable method to synthesize nanoparticles that can be utilized to prepare dense, high strength, and high toughness dispersion ceramics consisting of 47.6 vol% α-Al_2_O_3_ and 52.4 vol% t-ZrO_2_ by SPS. At first sight this seems quite astonishing because the CoLAVA nanoparticles consist of t-ZrO_2_ and transition alumina phases which are known to hinder a complete densification during sintering^18^. To understand this unusual behaviour it is necessary to have a closer look on the composition of the nanoparticles, their phase distribution, and the phase evolution during heat treatment as well as on specific peculiarities during the densification of alumina and zirconia ceramics by SPS.

**Figure 5:**
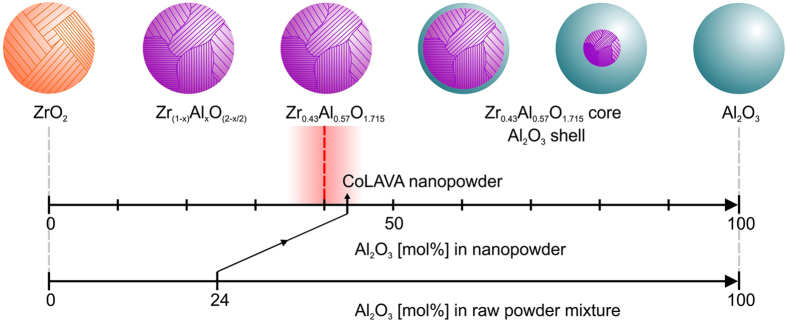
Composition and phase distribution of CoLAVA alumina/zirconia nanoparticles in dependence on the alumina content of the raw powder mixture.

Results from thermoanalyses, Fourier transform infrared (FTIR) spectroscopy, XRD, and TEM reveal that the CoLAVA nanoparticles mainly consist of t-ZrO_2_ and a small amount of transition alumina phases. Some of the particles exhibit a core/shell structure with a crystalline core and an amorphous shell. Sintered samples of the CoLAVA nanopowder consist of 47.6 vol% α-Al_2_O_3_ and 52.4 vol% t-ZrO_2_ of high crystallinity. This means that according portions of Al^3+^ and Zr^4+^ ions must have been present in the nanoparticles already after the CoLAVA process. Under thermodynamic equilibrium conditions there is no evidence for the formation of a solid solution in the zirconia-alumina system^19^. Alper presented a ZrO_2_-Al_2_O_3_ phase diagram according to which Al_2_O_3_ has a maximum of 7 mol% solubility in ZrO_2_ at 1885 °C^20^. However, in gas phase condensation processes like CoLAVA or flame pyrolysis the particle formation occurs within milliseconds^12^, i.e. far from thermodynamic equilibrium. For these conditions it was reported that a huge amount of up to 40 mol% Al_2_O_3_ can be incorporated into a t-ZrO_2_ defect crystal structure with the composition Zr_(1−x)_Al_x_O_(2−x/2)_^21^,^22^. It was suggested that Al^3+^ ions substitute Zr^4+^ ions by creating oxygen vacancies to maintain local charge balance^21^. Thus, nanoparticles with a threshold composition of Zr_0.43_Al_0.57_O_1.715_ are formed. At higher alumina concentrations - in our case 43 mol% - the exceeding alumina might form an amorphous shell around these defect crystals^23^. Due to the significantly higher melting and vaporization temperatures of zirconia compared with alumina, ZrO_2_ should condense and nucleate first from the gas phase followed by Al_2_O_3_^24^,^25^. The pre-condensed zirconia crystals subsequently act as nuclei for the heterogeneous nucleation of alumina. Figure 5 schematically illustrates the phase distribution within these nanoparticles. As mentioned earlier the difference in the alumina/zirconia ratio of raw powders and nanoparticles is reproducible and results from the higher vaporization rate of alumina when compared to zirconia.

**Figure 6:**
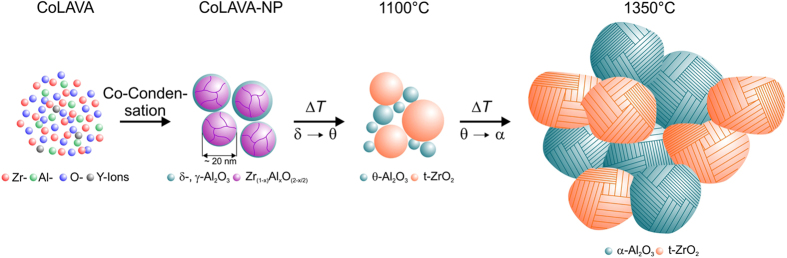
Gas phase condensation of alumina/zirconia hybrid nanoparticles in the CoLAVA process and their stepwise evolution at increasing sintering temperatures up to 1350 °C.

Heating the nanoparticles to 1100 °C leads to a phase transformation of the γ- and δ-alumina transition phases to θ-alumina which is in agreement with literature^26^. The Zr_(1−x)_Al_x_O_(2−x/2)_ defect structure seems to remain stable up to a temperature of 900 °C as can be seen by its constant domain size. Between 900 °C and 1100 °C the zirconia domains start to grow, and the thermal energy is finally used to separate θ-Al_2_O_3_ and t-ZrO_2_ (Fig. 6). Consequently, the XRD reflections of t-ZrO_2_ shift towards smaller diffraction angles. Only a small amount of alumina (<3 mol%) remains dissolved in the t-ZrO_2_ crystals^27^. Above 1300 °C θ-Al_2_O_3_ transforms to α-Al_2_O_3_. In pure alumina the θ to α transformation usually occurs at temperatures ranging from 1000 °C to 1200 °C^28^,^29^,^30^. The shift towards higher temperatures observed here is a consequence of the nanocrystallinity of the CoLAVA powder and is additionally supported by the stabilizing effect of zirconia. The θ- to α-Al_2_O_3_ transformation is considered to occur through a nucleation and growth process^31^. During thermal treatment the θ-Al_2_O_3_ crystallites grow and exceed a critical size of approximately 20 nm necessary for the exothermic formation of stable α-Al_2_O_3_^29^,^32^,^33^. Subsequently, the α-Al_2_O_3_ nuclei grow rapidly and form polycrystalline α-Al_2_O_3_ with crystallite sizes around 50 nm^32^,^34^. The phase transformation also attends a volume shrinkage (densities: *ρ*_θ*-alumina*_ = 3.60 g cm^−^^3^ and *ρ*_α*-alumina*_ = 3.99 g cm^−^^3^)^35^. However, under conventional sintering conditions it is hardly possible to obtain fully dense polycrystalline α-Al_2_O_3_ ceramics because the θ to α transformation is accompanied by the formation of vermicular microstructures consisting of a network of large pores^18^,^31^,^36^. This explains the residual porosity of 12% after conventional sintering at 1500 °C for 2 h. Hot pressing has been suggested as an appropriate method to limit the formation of vermicular pores by a pressure-induced particle rearrangement that cause impingement of the growing α-alumina colonies^18^. However, this route requires further doping elements to influence the γ to θ to α transformation^37^,^38^. On the other hand, the samples that were sintered by SPS at 1400 °C for 3 min in our study exhibit a density of 99% *φ*_*th*_. Some recent studies have shown that flash sintering allows the complete densification of certain ceramics within a few seconds at threshold conditions specified by the electric field and the furnace temperature^39^,^40^. In these cases sintering is accompanied by a sudden increase in the electrical conductivity of the specimen. ZrO_2_ ceramics flash sinter at 676 °C at a field of 1200 V cm^−1^ 39, whereas undoped, single-phase alumina remains immune to field assisted sintering at fields up to 1000 V cm^−1^ 40. Most recently, it was described that composites consisting of 50 vol% Al_2_O_3_ and 50 vol% ZrO_2_ flash sinter at a furnace temperature of 1060 °C under an electric field of 150 V cm^−1^ 41. However, in our case the electric field at 1400 °C was below 5 V cm^−1^ assuming a maximum voltage of 6 V, a minimum sample thickness of 3.6 mm (including graphite layers), and an effective voltage ratio of 0.3 for a SPS mold. Therefore, flash sintering can be excluded. Instead, it seems that the high pressure of 80 MPa that was applied during SPS in our case was most essential for the complete densification of the samples. This complete densification is a prerequisite to achieve excellent mechanical properties of technical ceramics. However, solely this could not explain the outstanding mechanical properties of ZrO_2_/Al_2_O_3_ ceramics sintered from the CoLAVA nanopowder by SPS. In particular the flexural strength of 1500 MPa is far beyond the state of the art. This high strength value can be attributed to comparatively small sizes of the ZrO_2_ (216 nm) and Al_2_O_3_ (270 nm) grains and a very homogeneous distribution of the dispersed phases after sintering. Both findings significantly differ from the results obtained for ceramics sintered from the WM reference powder. These ceramics exhibit larger grain sizes comparable to those described in literature for ZTA ceramics^2^,^7^ and a distinct tendency for the aggregation of both the Al_2_O_3_ grains and the ZrO_2_ grains. The fracture toughness (Table 1) of the CoLAVA nanopowder derived ZrO_2_/Al_2_O_3_ ceramics is 45% higher than *K*_*Ic*_ of the WM reference ceramics. However, it is only in the range of what is found in literature for ATZ and ZTA. The t- to m-ZrO_2_ transformability (Table 1) of our ZrO_2_/Al_2_O_3_ ceramics derived from the WM and the CoLAVA powder is clearly below the level of 77% we achieved for ZrO_2_ ceramics stabilized with 2 mol% Y_2_O_3_^14^. The lower coefficient of thermal expansion α of α-Al_2_O_3_ (*α*_*(300 K – 800 K)*_ = 6.6 × 10^−6 ^K^−1^) compared with yttria stabilized ZrO_2_ (*α*_*(300 K – 2000 K)*_ = 9.8 × 10^−6 ^K^−1^) is the reason for the tensile residual stress in ZrO_2_/Al_2_O_3_ ceramics. In the WM ceramic this stress acts non-uniformly due to the aggregation of the ZrO_2_ grains causing an increased partial transformation of t-ZrO_2_ during cooling down from the sintering temperature. Consequently, this reduces its transformability during the fracture process compared with the CoLAVA ceramics (Table 1). The superior transformability of the CoLAVA ceramic (Table 1) is also related to the reduced content of Y_2_O_3_ in the zirconia grains as measured by EDS. This could be due to the formation of the CoLAVA nanoparticles from the gas phase. During their condensation yttria is incorporated into the transition alumina phases. Heated above 1300 °C these alumina phases transform to α-Al_2_O_3_. However, yttria is not soluble in corundum and is segregated again. Actually, two weak reflections appear at 2*θ* = 29.2° and 48.6° in the diffractogram of the CoLAVA nanopowder sintered at 1350 °C (Fig. 3a) which correspond to the most intense reflections of Y_2_O_3_.

LTD resistance of the CoLAVA nanopowder derived ZrO_2_/Al_2_O_3_ ceramics is excellent and far beyond the levels that have been achieved for typical 3Y-TZP (3 mol% yttria stabilised tetragonal zirconia polycrystals) ceramics^14^ and yttria stabilised ZrO_2_/Al_2_O_3_ composites with a zirconia content beyond 25 wt% 42. Significant ageing followed by microcracking was noted at Al_2_O_3_ grain boundaries for ZrO_2_ contents exceeding a percolation limit of 16 vol% causing pathways for water diffusion from the surface towards the bulk^43^. For WM ceramics a first gradual increase up to 10 vol% of the monoclinic zirconia phase was observed after two hours and they exhibited a more rapid increase up to 40 vol% m-ZrO_2_ after 20 h of ageing treatment time. A degradation plateau was observed after 25 h. This behaviour was related to the presence of aggregated zirconia grains which act as further nucleation sites for the tetragonal-to-monoclinic transformation. However, a very limited ageing was observed for Al_2_O_3_/ZrO_2_ ceramics derived from the CoLAVA powder. There are several factors that might retard the degradation: The gas phase condensation of the CoLAVA nanoparticles proceeds fast and far from thermodynamic equilibrium. Hence, alumina is incorporated into zirconia during their co-condensation resulting in the formation of the defect structure Zr_(1−x)_Al_x_O_(2−x/2)_. Even after sintering some Al^3+^ remain dissolved in zirconia. These Al^3+^ ions now directly stabilize the tetragonal structure of zirconia instead of the Y^3+^ ions. Furthermore, Al_2_O_3_ that was initially dissolved segregates at the zirconia grain boundaries during sintering. Thus, it can effectively contribute to the improved degradation resistance as it was observed for Al_2_O_3_ doped Y-TZP ceramics^44^. Additionally, the homogeneously distributed alumina grains act as a constraint to the zirconia grains, retaining t-ZrO_2_ in a metastable state and making the material highly resistant to hydrothermal degradation.

The results of our study showed that the laser co-vaporization of mixed ZrO_2_ and Al_2_O_3_ raw powders followed by SPS of the obtained nanopowder is a highly suitable method to achieve very strong and tough dispersion ceramics with a high LTD resistance. In future investigations it seems to be promising to optimize the materials properties by adjusting the resulting ZrO_2_/Al_2_O_3_ ratios towards those of classical ATZ or ZTA ceramics. Furthermore, the yttria stabilization could be omitted. The obtained results suggest that for CoLAVA nanopowder derived Al_2_O_3_/ZrO_2_ dispersion ceramics a further stabilization is not necessarily required because the t-ZrO_2_ phase is stabilized by the incorporation of Al^3+^ ions in addition to strain effects of the alumina matrix due to the homogeneous distribution of the alumina and zirconia grains and their narrow size distribution.

## Methods

### Raw materials

Commercially available powders were used as raw materials: (1) tetragonal zirconia polycrystals (3Y-TZP, 3 mol% Y_2_O_3_; TZ-3YS-E, Tosoh Corp., Tokyo, Japan) with an average particle size *d*_*50*_ = 0.26 μm, (2) yttria-free monoclinic zirconia powder (TZ-0, Tosoh Corp., Tokyo, Japan) with an average particle size *d*_*50*_ = 0.30 μm, and (3) corundum (α-Al_2_O_3_; A16SG, Alcoa, USA) with an average particle size *d*_*50*_ = 0.53 μm.

### Materials processing

A zirconia powder with an overall yttria content of 2 mol% (2Y-TZP) was dry mixed from corresponding portions of 3Y-TZP and TZ-0^14^. In order to obtain a powder mixture containing 20 mass% of corundum appropriate quantities of this 2Y-TZP powder and the α-Al_2_O_3_ raw powder were mixed. Mixing was conducted in a polyethylene bottle with zirconia balls (diameter 1 mm, volume fraction 10%) in a multidirectional mixer (24 h at 150 rpm). From the zirconia-alumina mixture hybrid nanoparticles were prepared by using the CoLAVA method. For this purpose the mixture was vaporized applying pulsed CO_2_ laser radiation (wavelength 10.59 μm, pulse length 1 ms, pulse frequency 200 Hz, average radiation power 730 W, pulse peak power 3.5 kW, focus diameter 1 mm) and air as the process gas (flow rate in the zone of vaporization 2 m^3 ^h^−1^, total flow rate 14.5 m^3 ^h^−1^). Pulsed laser radiation was applied in order to narrow the particle size distribution and to minimize the fraction of primary particles firmly bonded by solid-state bridges^12^. The alumina and zirconia proportions in the CoLAVA nanopowder were evaluated from SEM micrographs of sintered (SPS), polished, and thermally etched specimens (Fig. 4b,d) by analysing the dark (Al_2_O_3_) and bright areas (ZrO_2_) with an image processing program (ImageJ 1.48 v, W. Rasband, National Institutes of Health, USA).

For comparison, a mixture of 62.6 mass% 2Y-TZP and 37.4 mass% α-Al_2_O_3_ raw powders was conventionally wet processed in distilled water with an alkali-free organic polyelectrolyte as surfactant. The wet mixture was homogenized by milling in a polyethylene bottle with zirconia balls (diameter 1 mm, volume fraction 10%, 24 h at 150 rpm) and then dried at 90 °C for 12 h. The resulting powder was ground in an agate mortar and subsequently passed through a 75 μm sieve in order to obtain the WM reference powder.

Compaction of the WM and CoLAVA powders was performed by using SPS (HP D 25, FCT Systeme GmbH, Frankenblick, Germany) at an impressed voltage of 4 V to 6 V in vacuum at 1400 °C applying a heating rate of 600 °C min^−1^ and an uniaxial pressure of 80 MPa. The final temperature and pressure were maintained for 3 min. The sintered specimens had diameters of 20 mm and 50 mm and a thickness of 2–4 mm.

### Characterization

#### Morphology, particle size distribution, and specific surface area of the CoLAVA nanopowder

Morphologic properties of the CoLAVA nanoparticles were evaluated by TEM (JEM 3010, JEOL Ltd., Tokyo, Japan, accelerating voltage 300 kV). For this purpose a small amount of the nanopowder was dispersed in ethanol, and drops of this suspension were deposited on a TEM grid (perforated carbon film on copper mesh, Plano GmbH, Wetzlar, Germany). The particle diameter distribution was determined from TEM micrographs^45^ by measuring the diameters of about 900 nanoparticles. From these data the percentage density distribution of the particle diameters on number basis *q*_*0*_ was compiled. The measured distribution was fitted with a logarithmic normal distribution in order to obtain the corresponding geometric mean particle diameter *μ*_*g*_(*q*_*0*_)^45^. The cumulative distribution of the particle diameters *Q*_*0*_ was fitted with a sigmoid function to obtain the characteristic particle diameters *d*_*10*_, *d*_*50*_, and *d*_*90*_.

The Brunauer-Emmett-Teller method (BET) was used for measuring the specific surface area *S*_*BET*_ of the CoLAVA nanopowder (Autosorb Automated Gas Sorption System with Autosorb Version 1.16, Quantachrome Instruments Corp., Boynton Beach, FL, USA). For this purpose the powder sample was dried and degassed at 350 °C for 5 h.

#### Thermoanalyses of the CoLAVA nanopowder

The phase transformations of the CoLAVA nanopowder were examined using DTA (NETZSCH STA 409 C/CD, NETZSCH-Gerätebau GmbH, Selb, Germany). For this purpose the nanopowder (170 mg) and a reference corundum powder (NETZSCH alumina, NETZSCH-Gerätebau GmbH, Selb, Germany) were filled into alumina crucibles. Both crucibles were heated up in air from room temperature to 1445 °C applying a heating rate of 5 °C min^−1^.

Shrinkage behaviour and dynamic sintering of green compacts of the CoLAVA nanopowder were investigated using a high-temperature horizontal dilatometer (DIL 802, BÄHR-Thermoanalyse GmbH, Hüllhorst, Germany) at a heating rate of 5 °C min^−1^ in air up to 1500 °C. The dwelling time at maximum temperature was 2 h.

#### XRD characterization

XRD measurements (D8 diffractometer, Bruker AXS Inc., Madison, WI, USA, Cu-Kα radiation, wavelength 1.5405981 Å, accelerating voltage 40 kV, beam current 30 mA) of the WM and CoLAVA powders as well as of annealed and sintered samples were performed at diffraction angles 2*θ* ranging from 20° to 70° (step scanning mode, step size 0.03°, scan speed 3.46° min^−1^). Qualitative analyses of the crystal phases were conducted using the following powder diffraction files: ICDD-PDF 01-083-0113 (t-ZrO_2_), ICDD-PDF 00-024-1165 (m-ZrO_2_), ICDD-PDF 00-046-1212 (α-Al_2_O_3_), ICDD-PDF 00-023-1009 (θ-Al_2_O_3_), ICDD-PDF 00-046-1215 (δ-Al_2_O_3_), and ICDD-PDF 00-050-0741 (γ-Al_2_O_3_). The mass fraction *X*_*m*_ of m-ZrO_2_ was evaluated using equation (1) 46:


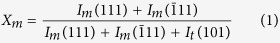


where *I*_*t*_ and *I*_*m*_ represent the integrated intensities (areas under the reflections) of the tetragonal (101)_t_ as well as the monoclinic (111)_m_ and 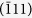
_m_ reflections. The volume fraction *V*_*mtot*_ of m-ZrO_2_ was calculated using equation (2) 47:


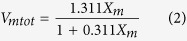


#### FTIR analyses of the CoLAVA nanopowder

FTIR spectra of the CoLAVA nanopowder as-prepared and after sintering at different temperatures from 500 °C to 1350 °C were measured (IFS 66v/S spectrometer, Bruker AXS Inc., Madison, WI, USA) in the wavenumber range 200 cm^−1^ to 1200 cm^−1^ (transmission mode, resolution 2 cm^−1^, 120 scans per sample). For this purpose KBr pellets (diameter 1.0–1.3 mm) of each powder sample were prepared using a uniaxial press. The vibrational bands of the FTIR spectra were assigned according to Boumaza *et al.*^48^.

#### ICP-OES analyses of calcined nanopowders

The distribution of Al, Zr, Y, and Hf in the CoLAVA nanopowder after calcination at 1350 °C was characterized by ICP-OES analyses (Agilent 720, Agilent Technologies, Santa Clara, CA, USA) after leaching in HCl. A custom designed charge-coupled detector (CCD) provided true simultaneous measurement, full wavelength coverage from 167 nm to 770 nm and fast read-out enabling short sample analysis times. The CCD detector has pixels arranged in continuous angled arrays that are matched exactly to the two-dimensional image from the Echelle polychromator. The source was powered by a radiofrequency generator operating at 40.48 MHz. The analyses were repeated five times. The results are given with their standard deviations.

#### Microstructure of sintered specimens

The sintered specimens were polished to 1 μm finish and thermally etched at 1350 °C for 30 min. The microstructure of gold coated samples was studied by SEM (AURIGA 60 FIB-SEM, CrossBeam Workstation, Carl Zeiss Microscopy GmbH, Jena, Germany). The average sizes of at least 150 alumina and zirconia grains per specimen were determined from SEM micrographs by using the linear intercept method^49^. The yttria distribution in ZrO_2_ grains on polished and thermally etched surfaces of sintered specimens was semi-quantitatively evaluated by EDS. In order to obtain well-resolved Y-Kα and Zr-Kα peaks the spectra were measured (Noran System SIX microanalysis system, Thermo Electron Corp., Waltham, MA, USA) applying an accelerating voltage of 20 kV, a beam current of 12 μA, and a total acquisition time of 5 min. 100 random points were analysed and the average percentage mole fraction of yttria was determined with an error of ±0.4 mol%. Bulk densities of the sintered specimens were determined by using the Archimedes method in water.

#### Mechanical properties of sintered specimens

The biaxial flexural strength was measured by using the piston-on-three-ball method (ISO 6872 standard). For this purpose disc specimens (diameter 20 mm, thickness 1.7 mm) were polished on one side and placed on three balls equispaced on a circle (diameter 10 mm) with the polished surface as the tensile side. A piston positioned above the centre of the three ball support applies a load to the unpolished side producing a biaxial flexural loading condition. The tests were performed at room temperature using a 5 kN universal testing machine (AutoGraph AG-X, Shimadzu Corp., Tokyo, Japan) with a piston speed of 1 mm min^−1^ until failure occurred. In order to obtain the average strength and elastic modulus, 12 specimens of each composition were tested. Details of data collection and calculation procedures have been reported elsewhere^50^.

The fracture toughness was measured by using single edge notched beams (SENB, dimension 3 mm × 4 mm × 45 mm). The tests were performed at room temperature using the 5 kN universal testing machine at a crosshead speed of 0.5 mm min^−1^ with a span of 40 mm. Notches were introduced by using a diamond blade saw. This method and the calculation of the fracture toughness have been reported elsewhere^51^.

The Vickers hardness of polished specimens was determined by microindentation with a diamond indenter (Leco 100-A, Leco Corp., St. Joseph, MI, USA). 10 indentations per sample were carried out under a 98 N load at an indentation time of 10 s. The magnitude of *HV* was calculated according to:


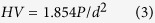


where *P* is the applied load (in N) and *d* the diagonal length (in mm).

#### Low temperature degradation of sintered specimens

Accelerated hydrothermal ageing was performed in an autoclave (Microclave 4001404, J.P. Selecta S.A., Barcelona, Spain) at 134 °C under a pressure of 200 kPa for up to 30 h. The sintered specimens were placed into the autoclave and left in a steam atmosphere. The LTD at predefined times was assessed by monitoring changes of the surface content of m-ZrO_2_ by means of XRD.

## Additional Information

**How to cite this article**: Bartolomé, J. F. *et al.* New ZrO_2_/Al_2_O_3_ Nanocomposite Fabricated from Hybrid Nanoparticles Prepared by CO_2_ Laser Co-Vaporization. *Sci. Rep.* **6**, 20589; doi: 10.1038/srep20589 (2016).

## Figures and Tables

**Table 1:** Densities and mechanical properties of the ceramic specimens sintered by SPS from the WM powder and the CoLAVA nanopowder as well as volume fractions of tetragonal “t” and monoclinic “m” zirconia in polished and fractured surfaces of the ceramic specimens and the resulting transformabilities of tetragonal zirconia.

Specimen	Density [% *φ*_*th*_]	Elastic modulus *E*[GPa]	Flexural strength σ_*f*_ [MPa]	Hardness *HV*[GPa]	Fracture toughness *K*_*Ic*_ [MPa m^1/2^]	Volume fractions of t- and m-ZrO_2_ [vol%]				Transformability of t-ZrO_2_ *V*_*trans*_
						**Polished**		**Fractured**		
						**t**	**m**	**t**	**m**	
WM	98	278	1100 ± 70	13.8 ± 0.4	4.7 ± 0.3	94	6	85	15	9
CoLAVA	99	279	1500 ± 30	14.9 ± 0.3	6.8 ± 0.2	98	2	64	36	34
